# Globulin and albumin to globulin ratio precisely diagnose periprosthetic joint infection and determine the timing of second-stage reimplantation

**DOI:** 10.1186/s13018-021-02899-0

**Published:** 2022-01-06

**Authors:** Guangqian Shang, ZhiXuan Fei, Hao Xu, Yingzhen Wang, Shuai Xiang

**Affiliations:** Department of Joint Surgery, The Affiliated Hospital of Qingdao University, Qingdao, 266000 Shandong China

**Keywords:** Periprosthetic joint infection, Platelet, Globulin, Reimplantation

## Abstract

**Background:**

Periprosthetic joint infection (PJI) is one of the most challenging complications of total joint arthroplasty (TJI). An early and accurate diagnosis of PJI is associated with better treatment outcomes. However, whether the platelet-related markers and globulin-related markers can be used to assist the diagnosis of PJI remains elusive.

**Methods:**

A total of 206 patients who underwent revision hip or knee arthroplasty in our institution were divided into two groups: 79 patients in PJI group and 127 patients in aseptic failure group. The levels of erythrocyte sedimentation rate (ESR), C-reactive protein (CRP), platelet-related markers including platelet count (PLT), mean platelet volume (MPV), plateletcrit (PCT) and PLT to MPV ratio (PMR) and globulin-related markers such as globulin (GLB), albumin to globulin ratio (AGR) and PLT to AGR ratio were compared. The diagnostic value was measured using area under the curve (AUC) after constructing receiver operating characteristic (ROC) curves. The potential of each marker for determining the timing of second-staged reimplantation was also evaluated.

**Results:**

Significantly increased levels of ESR, CRP, PLT, PCT, PMR, GLB and PLT to AGR ratio were identified in PJI group, while decreased levels of MPV and AGR were also found. The diagnostic values of all platelet-related markers and GLB were considered as fair, and good diagnostic values of AGR and PLT to AGR ratio were found, which were comparable to those of ESR and CRP. The levels of GLB and AGR can also be used to predict negative culture result and the timing of second-stage reimplantation.

**Conclusions:**

Globulin and albumin to globulin ratio were found to have good diagnostic values for PJI, and they can precisely predict the culture results and persistent infection.

## Introduction

Periprosthetic joint infection (PJI) and aseptic failure (AF) following total knee arthroplasty (TKA) and total hip arthroplasty (THA) are two major causes of the revision procedures [1, 2]. With a reported incidence of 0.5–2% after total joint arthroplasties, PJI has been regarded as one of the most challenging complications. The treatment of PJI is extremely different from that of AF with regard to a prolonged therapy, additional antibiotic use and failure in preserving the original implant [3]. A delayed diagnosis or even misdiagnosis will cause disastrous consequences. However, on some occasions, similar symptoms and signs such as chronic pain, joint swelling and elevated levels of inflammatory markers are presented in both AF and PJI patients. Normal plasma levels of traditional inflammatory markers are also observed in PJI patients infected with specific pathogens, making it difficult to distinguish PJI from AF [4]. As a result, an early and accurate diagnosis of PJI is extremely important to determine the final outcomes.

The introduction of the definition and diagnostic criteria by the Musculoskeletal Infection Society (MSIS) dramatically improved the diagnostic accuracy and treatment outcomes of PJI [5]. The modification of the MSIS criteria further enhances the sensitivity and specificity of PJI [6, 7]. A notable change in the 2018 definition of MSIS criteria is that the introduction and emphasize of D-Dimer in the diagnosis of PJI. D-Dimer (> 860 ng/mL) has been regarded as important as the traditional inflammatory marker, C-reactive protein (CRP), indicates the increasing attention on the linkage between coagulative markers and PJI. Recently, a series of studies, including our previous study with the same series of patients, have demonstrated the diagnostic value of plasma fibrinogen for PJI is equivalent to that of CRP or erythrocyte sedimentation rate (ESR) and is far better than that of D-Dimer [8–11]. These findings further promote the potential roles of the fibrinolytic markers in the diagnosis of PJI. Despite the fibrinolytic markers, several platelet-related markers, such as platelet count (PLT), mean platelet volume (MPV) and plateletcrit (PCT), have also been found to fluctuate during inflammatory and infection [12, 13]. Recent research has reported that the migrating platelets collect and bundle the microbial invaders, boost the activity of phagocytes and activate innate immune responses during infection [14]. Three studies have also investigated the possibility of platelet and its associated markers in diagnosing PJI, demonstrating a poor to fair diagnostic value [15–17]. In addition, evidences have also correlated the level and composition of serum proteins such as albumin (ALB) and globulin (GLB) with septic failure after joint arthroplasty [18, 19]. However, the number of the studies regarding these topics is still limited and further validation of the value of these markers for diagnosing PJI is needed.

The golden standard of the treatment protocol for PJI is the second-stage revision, including implant removal, thorough debridement and implanting of antibiotic-loaded spacer at first stage, systemic antibiotic treatment for six to eight weeks and a second-stage reimplantation [9]. Therefore, accurately predicting the complete control of infection is of importance to determine the timing of the second-stage reimplantation. Previous studies have discussed the possibility of ESR, CRP, D-Dimer and fibrinogen to identify residual infection after prior treatment [9, 20, 21]. As several newly emerging markers, whether the globulin-related markers and platelet-related markers could predict the persistent infection is still largely unknown.

In this study, we aimed to (1) explore the potential value of globulin-related markers and platelet-related markers for diagnosing PJI; (2) investigate the value of these markers for predicting the residual infection and the timing of second-stage reimplantation.

## Materials and methods

After obtaining the approval by the review board of our institution, a single-center, retrospective study was conducted to review the medical records of patients who had received revision knee or hip arthroplasty from August 2013 to December 2020. A total of 258 patients met the initial criteria and was included. A re-evaluation was then performed to exclude patients with reasons that may affect the platelet-related markers. As a result, 19 patients with periprosthetic fracture, six patients with insert dislocation, five patients with inflammatory diseases, one patient with recurrency of giant cell tumor and 16 patients with recent use of anticoagulants due to atrial fibrillation or venous thromboembolism (VTE) were excluded. We further excluded 5 patients with a diagnosis of PJI but only meet one to two minor criteria of the Musculoskeletal Infection Society (MSIS). At last, the remaining 206 patients were divided into two groups. The PJI group contained 79 patients, and 127 patients were in the aseptic failure group. Detailed information is displayed in Table 1. The demographic data, results of laboratory tests and the results of follow-up of the patients were recorded. The laboratory test was examined on the day of admission, and we recorded PLT, MPV, PCT, ALB, GLB and albumin to globulin ratio (AGR), as well as the perioperative results of pathogen culture. We also calculated the PLT to MPV ratio (PMR) and PLT to AGR ratio as two other markers. Notably, the comparison between the fibrinolytic markers and traditional inflammatory markers (CRP and ESR) of the same series of patients was also investigated and reported in the other study [11].

**Table 1:** Demographic characteristics of the included patients

	PJI group (*n* = 79)	AF group (*n* = 127)	*P* value
Gender			0.153
Male	44	57	
Female	35	70	
Age	68.13 ± 12.61	69.86 ± 10.46	0.309
BMI (kg/m^2^)	25.99 ± 3.59	25.11 ± 3.79	0.096
Affected joint			
Knee	62	54	0.003
Hip	37	73	

PJI, periprosthetic joint infection; AF, aseptic failure; BMI, body mass index

### Statistical analysis

SPSS version 23.0 (IBM Inc., Armonk, NY) was used to perform all statistical analyses. Data were presented with means ± standard deviations (SD) for continuous variables and frequencies or constituent ratios for categorical variables. Mann–Whitney test was used for comparing unpaired continuous variables and Wilcoxon’s signed-rank test for paired data. Statistical significance was set at *P* < 0.05. Receiver operating characteristic (ROC) curves of the biomarkers were generated for calculating the areas under the curves (AUCs), 95% confidence interval (CI), sensitivity and specificity. The AUC value determined the diagnostic value: excellent (0.990–1.0), good (0.800–0.899), fair (0.700–0.799), poor (0.600–0.699) and having no discriminatory capacity (0.500–0.599). The comparison between two AUCs was carried out using the method provided by Hanley and McNeil [22]. The optimal cutoff values of the biomarkers were then calculated based on the Youden index, and positive predictive value (PPV) and negative predictive value (NPV) were also calculated according to the optimal cutoff values. Figures were drawn using GraphPad Prism software (version7.0; GraphPad Software Inc., San Diego, CA).

## Results

### Diagnostic values of the markers for PJI

**Fig. 1:**
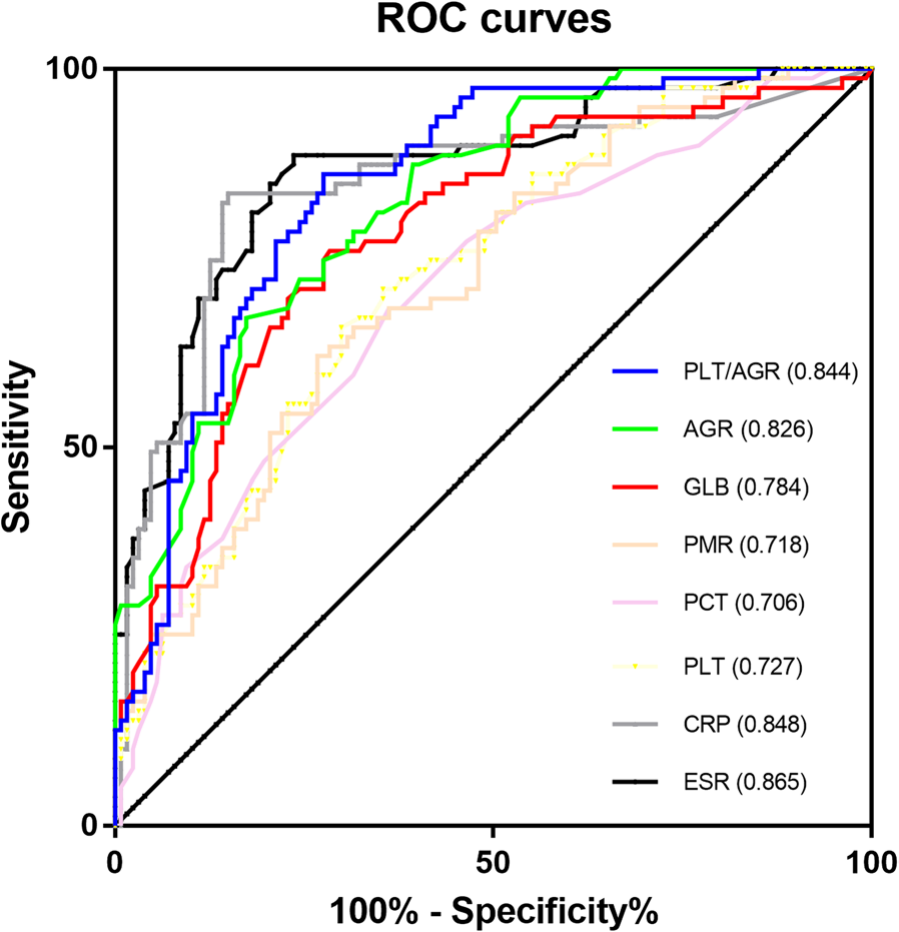
The ROC curves of plasma CRP, ESR, PLT, PCT, PMR, GLB, AGR and PLT to AGR ratio. CRP, C-reactive protein; ESR, erythrocyte sedimentation rate; PLT, platelet count; PCT, plateletcrit; PMR, PLT to MPV ratio; MPV, mean platelet volume; PMR, PLT to MPV ratio; GLB, globulin; AGR, albumin to globulin ratio; ROC, receiver operating characteristic curve

Age, gender and BMI of the patients were not statistically different between the two groups. However, more knees were affected in PJI group than in AF group (Table 1). Levels of PLT, MPV, PCT, PMR, ALB, GLB, AGR and PLT to AGR ratio were recorded and calculated. Plasma levels of two traditional inflammatory markers (ESR and CRP) were also screened. In PJI group, significantly higher PLT (288.82 ± 97.79 vs. 221.83 ± 63.52, *P* < 0.001), PCT (0.28 ± 0.08 vs. 0.23 ± 0.10, *P* < 0.001), PMR (29.91 ± 11.96 vs. 22.11 ± 11.26, *P* < 0.001), GLB (33.46 ± 5.84 vs. 27.48 ± 5.16, *P* < 0.001) and PLT to AGR ratio (290.91 ± 141.43 vs. 166.68 ± 66.07, *P* < 0.001) were identified, while the MPV (9.86 ± 0.92 vs. 10.22 ± 0.97, *P* < 0.001) and AGR (1.06 ± 0.22 vs. 1.40 ± 0.27, *P* < 0.001) were significantly decreased (Table 2). Although the level of ALB of PJI group was slightly lower than that of AL group (35.51 ± 5.36 vs. 36.71 ± 5.21), statistical significance was not found (*P* = 0.086, Table 2). ROC curves of CRP, ESR, PLT, PCT, PMR, ALB, GLB, AGR and PLT to AGR ratio were generated to evaluate their diagnostic value and the markers with an AUC more than 0.700 were displayed (Fig. 1 and Table 3). For PLT, the optimal cutoff value was 241.5 × 10^9^/L (sensitivity: 65.8%, specificity: 70.1%, PPV: 57.8%, and NPV: 76.7%), with an AUC of 0.727 (95% confidence intervals [CI]: 0.657–0.796). For PCT, the optimal cutoff value was 0.236, with a sensitivity of 68.4%, a specificity of 63.8%, a PPV of 54.0%, a NPV of 76.4% and an AUC of 0.706 (95% CI 0.633–0.779). The optimal cutoff value and AUC of PMR were 25.31 (sensitivity: 62.0%, specificity: 73.2%, PPV: 59.0%, and NPV: 75.6%) and 0.718 (95% CI 0.648–0.788), respectively. The AUCs of the platelet-related markers were far lower than the AUCs of CRP and ESR, which were 0.848 and 0.865, respectively. However, when combining the above optimal cutoff values of platelet-related markers with traditional inflammatory markers, we found a remarkable improvement in sensitivity, specificity, PPV and NPV for diagnosing PJI (Table 4). Meanwhile, we also identified potential value of globulin-related markers for diagnosing PJI. The AUC of GLB was 0.784 (95% CI 0.719–0.849), and the sensitivity, specificity, PPV and NPV were 80.0%, 71.7%, 75.9% and 72.4%, respectively. For AGR, the AUC was 0.826 (95% CI 0.770–0.880), with a sensitivity of 67.1%, a specificity of 86.7%, a PPV of 67.1%, a NPV of 80.3%. The AUC of PLT to AGR ratio was 0.844 (95% CI 0.791–0.898, sensitivity: 86.1%, specificity: 76.4%, PPV: 86.1% and NPV: 72.4%). The diagnostic value of GLB, AGR and PLT to AGR ratio was equivalent to that of ESR and CRP (Table 4).

**Table 2:** Comparison of the plasma levels of the markers

Markers	Range	PJI group	AF group	*P* value
CRP (mg/L)	< 5.00	36.95 ± 48.78	8.02 ± 26.09	< 0.001
ESR (mm/h)	< 30.00	43.27 ± 26.96	14.16 ± 11.51	< 0.001
PLT (× 10^9^/L)	125–350	288.82 ± 97.79	221.83 ± 63.52	< 0.001
MPV (fL)	7.4–11.0	9.86 ± 0.92	10.22 ± 0.97	0.006
PCT (%)	0.09–0.30	0.28 ± 0.08	0.23 ± 0.10	< 0.001
PMR	–	29.91 ± 11.96	22.11 ± 11.26	< 0.001
GLB (g/L)	20–40	33.46 ± 5.84	27.48 ± 5.16	< 0.001
ALB (g/L)	40–55	35.51 ± 5.36	36.71 ± 5.21	0.086
AGR	1.2–2.4	1.06 ± 0.22	1.40 ± 0.27	< 0.001
PLT/AGR	–	290.91 ± 141.43	166.68 ± 66.07	< 0.001

PJI, periprosthetic joint infection; AF, aseptic failure; ESR, erythrocyte sedimentation rate; CRP, C-reactive protein; PLT, platelet count; MPV, mean platelet volume; PCT, plateletcrit; PMR, PLT to MPV ratio; GLB, globulin; AGR, albumin to globulin ratio

**Table 3:** Diagnostic value of the markers for PJI

Markers	AUC (95% CI)	Youden index	Optimal cutoff value	Sensitivity (%)	Specificity (%)	PPV (%)	NPV (%)	*P*^a^	*P*^b^
CRP (mg/L)	0.848 (0.788–0.907)	0.686	8.15	83.5	85.0	77.6	89.3		
ESR (mm/h)	0.865 (0.813–0.918)	0.616	15.70	88.6	75.4	70	91.5		
PLT (× 10^9^/L)	0.727 (0.657–0.796)	0.359	241.5	65.8	70.1	57.8	76.7	0.009	0.002
PCT (%)	0.706 (0.633–0.779)	0.321	0.236	68.4	63.8	54.0	76.4	0.003	0.0005
PMR	0.718 (0.648–0.788)	0.353	25.31	62.0	73.2	59.0	75.6	0.006	0.001
GLB	0.784 (0.719–0.849)	0.476	30.65	80.0	71.7	75.9	72.4	0.157	0.049
AGR	0.826 (0.770–0.880)	0.398	1.165	67.1	86.7	67.1	80.3	0.594	0.308
PLT/AGR	0.844 (0.791–0.898)	0.585	188.9	86.1	76.4	86.1	72.4	0.937	0.586

PJI, periprosthetic joint infection; CI, confidence interval; AUC, area under the curve; PPV, positive predictive value; NPV, negative predictive value; ESR, erythrocyte sedimentation rate; CRP, C-reactive protein; PLT, platelet count; MPV, mean platelet volume; PCT, plateletcrit; PMR, PLT to MPV ratio; GLB, globulin; AGR, albumin to globulin ratio

^a^Compared with the AUC of CRP

^b^Compared with the AUC of ESR

**Table 4:** Combinational diagnostic value of the markers for PJI

Markers	Sensitivity (%)	Specificity (%)	PPV (%)	NPV (%)
PLT combined with inflammatory markers				
PLT and ESR	51.9	55.9	46.1	67.5
PLT or ESR	94.9	90.6	84.3	96.6
PLT and CRP	62.0	63.0	51.0	72.7
PLT or CRP	87.3	92.1	87.3	92.1
PCT combined with inflammatory markers				
PCT and ESR	63.3	57.5	48.1	71.6
PCT or ESR	93.7	91.3	87.1	95.9
PCT and CRP	64.6	52.0	45.5	70.2
PCT or CRP	87.3	90.3	82.1	91.8
PMR combined with inflammatory markers				
PMR and ESR	55.7	59.1	45.8	62.5
PMR or ESR	94.9	90.6	86.2	96.6
PMR and CRP	59.5	65.4	51.6	72.2
PMR or CRP	86.1	92.9	88.3	91.5

PJI, periprosthetic joint infection; CI, confidence interval; AUC, area under the curve; PPV, positive predictive value; NPV, negative predictive value; ESR, erythrocyte sedimentation rate; CRP, C-reactive protein; PLT, platelet; PCT, plateletcrit; PMR, PLT to MPV ratio; MPV, mean platelet volume

### The application of the markers for predicting culture results and second-stage reimplantation

In PJI group, there were 60 patients with culture-positive PJI and 19 with culture-negative PJI. When comparing the levels of the PLT, PCT, MPV and PMR between these two groups, no significance was found (Table 5). However, the level of GLB was significantly elevated and the level of AGR was significantly decreased in patients with culture-positive patients. To investigate the value of the markers for determining second-stage reimplantation, we also recorded the data of 42 patients who finished second-stage reimplantation with no reinfection after 2-years follow-up. The levels of PLT, MPV, PCT, PMR, ALB, GLB, AGR and PLT to AGR ratio before first-stage resection and before second-stage reimplantation were paired and analyzed. Compared with those before the first-stage resection and spacer implantation, the levels of PLT (221.53 ± 51.75 vs. 282.22 ± 71.56, *P* < 0.001), PCT (0.22 ± 0.05 vs. 0.27 ± 0.07, *P* < 0.001), PMR (22.16 ± 5.60 vs. 29.47 ± 8.37, *P* < 0.001), GLB (33.32 ± 5.70 vs. 27.73 ± 3.65, *P* < 0.001) and PLT to AGR ratio (280.21 ± 160.48 vs. 161.39 ± 46.23, *P* < 0.001) before the second-stage reimplantation were significantly decreased, while the MPV (10.06 ± 0.65 vs. 9.67 ± 0.73, *P* = 0.001) and AGR (1.05 ± 0.19 vs. 1.41 ± 0.21, *P* < 0.001) were significantly higher (Table 6).

**Table 5:** Plasma levels of all markers in patients with culture-positive and culture-negative PJI

Markers	Culture-positive PJI (*n* = 60)	Culture-negative PJI (*n* = 19)	*P* value
CRP (mg/L)	39.56 ± 51.78	28.73 ± 37.83	0.450
ESR (mm/h)	46.32 ± 28.35	33.68 ± 19.69	0.123
PLT (× 10^9^/L)	284.03 ± 105.21	303.93 ± 69.87	0.120
MPV (fL)	9.83 ± 0.92	9.95 ± 0.92	0.536
PCT (%)	0.28 ± 0.09	0.30 ± 0.06	0.080
PMR	29.53 ± 12.88	31.09 ± 8.60	0.175
GLB	35.05 ± 6.25	31.61 ± 3.89	0.036
AGR	1.02 ± 0.21	1.21 ± 0.23	0.014
PLT/AGR	298.21 ± 155.53	268.04 ± 80.83	0.939

PJI, periprosthetic joint infection; ESR, erythrocyte sedimentation rate; CRP, C-reactive protein; PLT, platelet; MPV, mean platelet volume; PCT, plateletcrit; PMR, PLT to MPV ratio; GLB, globulin; AGR, albumin to globulin ratio

**Table 6:** Plasma levels of all markers in patients with successfully controlled infection

Markers	Normal range	Pre-resection	Pre-reimplantation	*P* value
CRP (mg/L)	< 5.00	31.67 ± 36.76	3.40 ± 6.96	< 0.001
ESR (mm/h)	< 30.00	43.97 ± 25.19	10.82 ± 6.53	< 0.001
PLT (× 10^9^/L)	125–350	282.22 ± 71.56	221.53 ± 51.75	< 0.001
MPV (fL)	7.4–11.0	9.67 ± 0.73	10.06 ± 0.65	0.001
PCT (%)	0.09–0.30	0.27 ± 0.07	0.22 ± 0.05	< 0.001
PMR	–	29.47 ± 8.37	22.16 ± 5.60	< 0.001
GLB (g/L)	20–40	33.32 ± 5.70	27.73 ± 3.65	< 0.001
AGR	1.2–2.4	1.05 ± 0.19	1.41 ± 0.21	< 0.001
PLT/AGR	–	280.21 ± 160.48	161.39 ± 46.23	< 0.001

Wilcoxon’s signed-rank test for paired samples (*n* = 42)

PJI, periprosthetic joint infection; ESR, erythrocyte sedimentation rate; CRP, C-reactive protein; PLT, platelet; MPV, mean platelet volume; PCT, plateletcrit; PMR, PLT to MPV ratio; GLB, globulin; AGR, albumin to globulin ratio

## Discussion

In the present study, we successfully demonstrated fair to good diagnostic values of PLT, MPV, PCT, PMR, GLB, AGR and PLT to AGR ratio for diagnosing PJI. Meanwhile, they could also be used to predict the eradication of infection after prior treatment. Compared with those of ESR and CRP, the diagnostic values of GLB, AGR and PLT to AGR ratio were not inferior, indicating their potential usage for diagnosing PJI. Although the diagnostic value, sensitivity and specificity of PLT, PCT and PMR were worse when comparing with the traditional inflammatory markers currently used for diagnosing PJI, the combination of the platelet-related markers with ESR and CRP could achieve surprisingly high sensitivity and specificity for diagnosing or ruling out PJI. Thus, the platelet indices could be regarded as adjunct tools.

Although the modification of MSIS criteria in 2018 significantly improved the diagnostic sensitivity of PJI, effort has never stopped to find out novel serum or synovial markers to facilitate diagnosis and treatment [6]. As a result, a series of markers and advanced technologies have been introduced into the clinical utility for diagnosing PJI. Despite plasma D-Dimer and the synovial α-Defensin, which have been already included into the modification of MSIS criteria, plasma fibrinogen and the synovial leukocyte esterase have also been intensively focused in recent years [8, 23]. In multiple studies, plasma fibrinogen has been regarded as a promising marker with equivalent diagnostic value to the traditional inflammatory markers, ESR and CRP [9, 16]. Similarly, we also found good diagnostic value of plasma fibrinogen for PJI, which was comparable to those of ESR and CRP. We further demonstrated the potential utility of plasma fibrinogen in predicting persistent infection before second-stage reimplantation. Although in our study D-dimer had a poor diagnostic value for PJI, it might be a useful marker for predicting negative culture results [11]. Compared with synovial markers, serum or plasma markers were less invasive and more economic friendly for the patients. Thus, other plasma markers or their combinations are also being investigated to explore their potential utility in PJI.

Serum albumin and globulin have been demonstrated to be implicated in diverse infection and inflammation [24]. Although albumin is initially considered as a marker reflecting nutritional condition, it is negatively regulated by the acute phase reactant and the association between hypoalbuminemia and septic failure after joint replacement has been discovered [18, 25]. Serum globulin mainly contains antibodies and inflammatory cytokines, and responds to inflammatory and infective reactions [26]. In this study, increased serum level of globulin and decreased AGR were found to be effective diagnostic markers for PJI; however, the diagnostic value of albumin was limited. Our results have also been corroborated by two other recent studies [19, 26]. However, in these two studies, the authors did not compare the diagnostic values of GLB and AGR with those of ESR and CRP. Here, we compared the ROC curves using the method described by Hanley and McNeil [22]. The AUC of AGR was not statistically different from those of ESR (*P* = 0.307) and CRP (*P* = 0.594). However, although no significance was found when comparing with CRP (*P* = 0.157), the AUC of GLB was significantly lower than that of ESR (*P* = 0.049). Particularly, AGR can be used as a convenient marker to distinguish PJI from AL since the AGR value of patients with PJI is lower than the normal range, while most AL patients presented with normal AGR.

It has long been noticed that platelets function as a vital regulator of innate immunity to diverse infection [27]. In sepsis, the direct or indirect crosstalk between platelets and bacteria induces platelet activation, aggregation and thrombus formation in the microvasculature, limiting the invasion and dissemination of the bacteria [28]. In addition, the interaction between platelets and leukocytes through membrane receptors, such as toll-like receptors (TLRs), contributes to the formation of platelet-leukocyte aggregate and triggers the bacterial phagocytosis [29]. However, only recently has the relationship between platelet-related markers and PJI been noticed. Paziuk et al. firstly reported the use of PLT and MPV for diagnosing PJI [15]. Instead analyzing the two markers independently, they combined them together by calculating PMR. The AUC of PMR was 0.69 and the sensitivity was 48.10%. When combined PMR with ESR and CRP, the AUC increased from 0.8749 to 0.8768. As a result, they considered PMR as an adjunct indicator of PJI. Later, Xu et al. investigated the potential value of PLT alone for diagnosing PJI and reported a fair diagnostic value at a cutoff threshold of 221 × 10^9^/L [16]. Another study conducted by Tirumala et al. used PMR for diagnosing PJI specifically after TKA. They reported a good diagnostic value of PMR, with a AUC of 0.85 [17]. In their study, PMR even outperformed CRP in sensitivity, while the specificity of PMR was slightly lower. Moreover, when combining PMR with ESR and CRP, the sensitivity, specificity, PPV and NPV were above or near 90%. The conclusions reached in these three previous studies were partly supported by our results. In our study, we also found significantly elevated averaged level of PLT and decreased averaged level of MPV. By calculating the AUC of ROC curve, the diagnostic values of PLT and PMR for PJI were considered as fair (AUC: 0.727 and 0.718, respectively), which were significantly lower than those of ESR and CRP. Compared with the results reported by Paziuk et al., our results showed better diagnostic value and sensitivity of PMR. However, the AUC, sensitivity, specificity, PPV and NPV of the ratio were inferior to those of CRP and ESR in our study. When combined the inflammatory markers with either PLT or PMR, the combinational diagnostic sensitivity and specificity reached 90%, which somehow supported the results reported by Tirumala et al. Despite the previously studied PLT and PMR, we investigated the utility of another platelet-related marker, plateletcrit (PCT), for diagnosing PJI. Increased level of PCT has been reported to be associated with infection and hospital mortality in intensive care unit patients [30, 31]. For the first time, we evaluated its possibility for diagnosing PJI. Although the AUC of PCT was the lowest among the platelet-related markers, it displayed better sensitivity than PLT and PMR. The combinational sensitivity and specificity of PCT and inflammatory markers were also somewhat equivalent to those of other platelet indices. Further, we intended investigate whether it would facilitate the diagnosis of PJI when taking both platelet function and serum globulin into account. Thus, we calculated the PLT to AGR ratio and we found the AUC of PLT to AGR ratio was slightly higher than that of AGR and comparable to those of ESR and CRP.

The most determinative and instructive examination for diagnosing PJI is pathogen culture. The culture results not only confirm the diagnosis but also determine the subsequent antibiotic selection. However, culture-negative PJI occurs in up to 40% of all patients [32]. The prediction of the negative culture results might help the surgeons turn to more advanced methods for assistance. In this study, the PLT, MPV, PCT, PMR, GLB, AGR and PLT to AGR ratio were compared between patients with positive culture results and negative culture results. We found significantly decreased GLB and increased AGR in culture-negative patients, indicating that the GLB and AGR had the potential to predict negative culture results. However, other markers failed to predict culture-negative PJI in our study. Nevertheless, by pairing the data before first-stage resection and second-stage reimplantation, we observed significantly decreased levels of PLT, PCT, PMR, GLB and PLT to AGR ratio, as well as increased level of MPV and AGR, in patients who had successfully controlled infection. In their study, Tirumala et al. have also demonstrated significant changes of the levels of platelet-related markers in a larger population before and after revision TKA specifically [17]. However, in our study, we firstly expanded the utility of platelet-related markers to both revision THA and TKA. Moreover, in accordance with previous literatures [9, 20], we analyzed paired data from same patient who fulfilled a 2-year follow-up after reimplantation with successful infection eradication, which was more persuasive to indicate that the platelet indices could become the referable tools when deciding the timing of second-stage reimplantation.

One of the major limitations of this study pertains to its retrospective design, resulting in inherent biases. In addition, the sample size in this single center is limited. To clarify the roles of platelet indices for diagnosing PJI, more prospective, multicentered studies are needed. For platelet-related indices, although the levels of the platelet indices were significantly different between PJI and AF patients, they failed to display better diagnostic outcomes than ESR and CRP. As a result, they can only act as adjunct tools for diagnosing PJI.

In conclusion, the globulin-related markers, such as GLB, AGR and PLT to AGR ratio, have equivalent diagnostic values for PJI compared with ESR and CRP. GLB and AGR also have the potential to predict negative culture results and determine the timing of second-stage reimplantation. For platelet-related markers, such as PLT, PCT and PMR, although they are easily and economic friendly to obtain, and have fair diagnostic values for PJI, no superiority was found when comparing with ESR and CRP.

## Data Availability

None.

## Abbreviations

PJI: Periprosthetic joint infection
AF: Aseptic failure
TKA: Total knee arthroplasty
THA: Total hip arthroplasty
MSIS: The Musculoskeletal Infection Society
ROC: Receiver operating characteristic
CI: Confidence interval
AUC: Area under the curve
PPV: Positive predictive value
NPV: Negative predictive value
ESR: Erythrocyte sedimentation rate
CRP: C-reactive protein
PLT: Platelet count
MPV: Mean platelet volume
PCT: Plateletcrit
PMR: PLT to MPV ratio
GLB: Globulin
AGR: Albumin to globulin ratio
